# The Distinct Biological Effects of 6-Hydroxy-L-Nicotine in Representative Cancer Cell Lines

**DOI:** 10.3390/molecules29235593

**Published:** 2024-11-26

**Authors:** Paula Alexandra Postu, Razvan Stefan Boiangiu, Marius Mihasan, Alexandru Bogdan Stache, Adrian Tiron, Lucian Hritcu

**Affiliations:** 1 Center for Fundamental Research and Experimental Development in Translation Medicine–TRANSCEND, Regional Institute of Oncology, 700483 Iasi, Romania; paula.postu@iroiasi.ro (P.A.P.); stache.bogdan@gmail.com (A.B.S.); 2 Department of Biology, Faculty of Biology, Alexandru Ioan Cuza University of Iasi, 700506 Iasi, Romania; razvan.boiangiu@uaic.ro (R.S.B.); marius.mihasan@uaic.ro (M.M.)

**Keywords:** 6-hydroxy-L-nicotine, nicotine, nicotinic receptors, cancer

## Abstract

6-hydroxy-L-nicotine (6HLN) is a nicotine (NIC) derivative with proven therapeutic potential in neurodegenerative disorders. Here, the impact of 6HLN on cell growth, migratory behavior, and inflammatory status of three different cancer cell lines (A549, MCF7, and U87) and two normal cell lines (16HBE14o and MCF10A) was investigated. In silico analyses were conducted to evaluate the binding affinity of 6HLN to nicotinic receptors (nAChRs) containing α9 and α5 subunits. The obtained in silico data revealed that 6HLN might act on the cholinergic system. Interestingly, the in vitro data showed the compound has cancer-stimulatory effects in U87 glioblastoma cells and cancer-inhibitory effects in MCF7 breast cancer cells. In A549 lung cancer cells, no changes were detected upon 6HLN administration. More importantly, 6HLN appears not to be deleterious for normal cells, with the viability of 16HBE14o pulmonary cells and MCF10A mammary cells remaining unchanged.

## 1. Introduction

Conventionally regarded as nerve-secreted chemicals, neurotransmitters are responsible for stimulatory or inhibitory signal transmission, ensuring intercellular communication and underlying fundamental and intricate biological functions, in both the central nervous system (CNS) and peripheral nervous system (PNS) [1]. Impaired neurotransmission has been generally correlated with the development of neurological and neurodegenerative disorders [2], yet paracrine and autocrine signaling via neurotransmitters have been recently acknowledged as a key component of human malignancies [3,4]. It has been shown that both nerve fibers and tumor cells actively participate in shaping an adaptive microenvironment that favors tumor growth. As such, the nerve fiber-derived neurotransmitters stimulate survival and proliferation of the tumor cells which are expressing specific neurotransmitter receptors, while the tumor cells are producing endogenous neurotransmitters as a response to various microenvironmental stimuli [5]. This type of nervous system–cancer crosstalk has been already observed in experimental models of prostate, gastric, pancreatic, skin, and breast cancers [6]. Moreover, a tissue-type dependency has been observed for different neurotransmitter systems. For instance, in both pancreatic and breast cancers, β-adrenergic signaling exerts growth-promoting effects, whereas cholinergic signaling suppresses tumor growth [7].

The main player in cholinergic signaling, acetylcholine (ACh) acts predominantly as a modulatory neurotransmitter in CNS and as an excitatory neurotransmitter in PNS, ensuring fast, but transient neurotransmission due to the prompt inactivation of the neurochemical by acetylcholinesterase (AChE) [8]. ACh released at the presynaptic level binds to its cognate fast-activating nicotinic (nAChRs) and slow-activating muscarinic (mAChRs) receptors [9]. Generally considered to be confined at the CNS (neuronal nAChRs) and at neuro-muscular junctions (muscle nAChRs), nAChRs have recently been found to be expressed by various types of cells, including cancer cells. Tumor cells express both neuronal and muscle nAChRs, yet only the function of neuronal nAChRs is currently being investigated in the oncological field [10]. Human neuronal nAChRs are pentameric assemblies of diverse α and β subunit combinations, the specific configuration of nAChR subunits having an essential role in regulating the function of the receptor [9]. In both CNS and cancer, the homomeric α7 receptors act as the main growth stimulatory nAChRs, whereas the heteromeric α4β2 receptors are the primary growth inhibitory nAChRs [11].

One of the main exogenous nAChR agonists is nicotine (NIC), which binds to the receptors with a greater affinity than ACh [12]. In cancer cells, it has been shown that NIC acts via α7 and α9 homomeric nAChRs stimulating proliferation and increasing aggressiveness [13,14]. The α5 constitutive subunit of heteromeric nAChRs, such as α3β4α5 and α3β2α5, also appears to be involved in promoting the growth stimulatory effects of NIC in human lung cancer specimens [15]. NIC-derivatives, such as N-nitrosonornicotine (NNN) and 4-(methylnitrosamino)-1-(3-pyridyl)-1-butanone (NNK), bind to nAChRs with much higher affinity than NIC and, similar to their parent compound, they act as protumorigenic agents [11]. In silico studies have shown that another NIC derivative—5-[(2S)-1-methylpyrrolidin-2-yl]pyridin-2-ol (6-hydroxy-L-nicotine or 6HLN), demonstrates a higher affinity for nAChRs compared to NIC, with research on its effects being limited to possible applications in neurodegenerative disorders, such as Alzheimer’s disease (AD) [16]. In different in vivo AD models, 6HLN overcame NIC shortcomings, being more potent in attenuating cognitive deficits, restoring redox balance, and reducing inflammation [16,17].

Alongside conventional approaches, manipulation of the cholinergic system has begun to be regarded as a promising strategy for controlling cancer progression. Most of the current research is focused on antagonizing nAChRs to reduce the survival of cancer cells [18]. Yet, Kolodziej et al. [19] reported that stimulation of nAChRs may also result in anti-proliferative effects. Therefore, this study focused on 6HLN, attempting to assess whether this nAChR agonist inhibits or, conversely, sustains cancer cell progression, in a similar manner to its parental compound, NIC. Considering that the effects of 6HLN have not been previously studied in cancer pathologies, all results obtained using 6HLN are discussed in contrast to the results obtained using NIC, a standard compound used to stimulate nAChRs.

## 2. Results and Discussion

### 2.1. α9,α3β2α5 and α3β4α5 nAChR Structures Prediction and In Silico Molecular Docking of 6HLN and NIC

It has been previously reported that both NIC and 6HLN bind to α7 nAChRs [16]. However, in the human cancer cell lines A549 (lung carcinoma), MCF7 (breast carcinoma), and U87 (glioblastoma), the expression of α7 subunits is low compared to the expression of α5 or α9 subunits [20,21,22]. Therefore, the binding potential of NIC and 6HLN to the homomeric α9 and heteromeric α3β2α5 and α3β2α5 subtypes of nAChRs was evaluated using in silico molecular docking. Since the 3D structure of these receptors has not yet been experimentally determined, AlphaFold2 multimer was used to model the structures of α9 (Supplementary Figure S1), α3β2α5 (Supplementary Figure S2), and α3β4α5 (Supplementary Figure S3) nAChRs. Following computational modeling, 25 structures were obtained and ranked based on confidence score expressed as predicted Local Distance Difference Test (pLDDT). For in silico molecular docking, the structure with the best pLDDT score for the residues involved in the formation of the NIC-binding site was chosen. The identification of these residues has been achieved through the alignment of the α9, α3, and α5 subunit sequence with the α4 sequence of the experimentally determined structure of α4β2 nAChRs; the same procedure was followed for β2 and β4 subunit sequences, which were aligned with β2 sequence of α4β2 nAChRs. Thus, seven common residues that are involved in the NIC-binding site formation in the α9 subunit have been identified, as well as seven residues in the α3 subunit and six residues in the α5 subunit (Supplementary Table S1). As for subunits β2 and β4 from the modeled structures, eight and six common residues were found (Supplementary Table S2). Except for two cysteine residues of the α5 subunit, all these residues obtained pLDDT scores above 80, suggesting a high level of confidence in the predicted NIC-binding site of α9, α3β2α5, and α3β4α5 nAChRs. Next, the binding potential of NIC and 6HLN to the modeled structures was evaluated. To validate the in silico docking procedures, the co-crystalized NIC molecule was removed from the α4-α4 and α4-β2 interfaces of the experimentally determined α4β2 nAChRs structure (PDB ID 6CNK) and (*S*)-NIC was docked in the binding pocket. The Root Mean Square Deviation (RMSD) was calculated and the ligand’s orientation with the native position was compared. The very good fit between computationally obtained (*S*)-NIC orientation and the one obtained experimentally (Supplementary Figure S4, RMSD of 0.23 Å at α4-α4 interface and 0.31 Å at α4-β2 interface respectively) indicates a reliable docking procedure.

**Figure 1:**
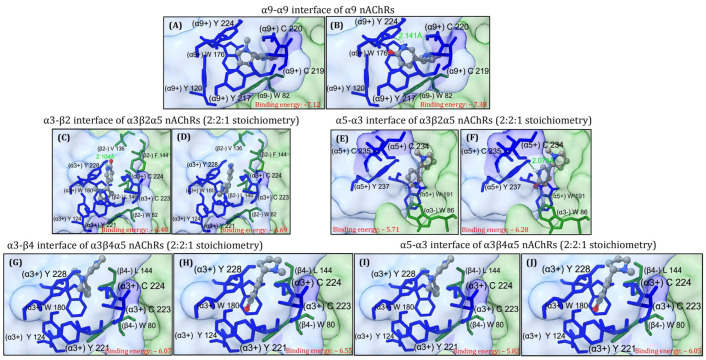
The best theoretical binding position of (s)-nicotine and (s)-6-hydroxynicotine at the α9-α9 interface of α9 nAChRs (**A**,**B**), α3-β2 (**C**,**D**) and α5-α3 (**E**,**F**) interfaces of α3β2α5 nAChRs (2:2:1 stoichiometry) and α3-β4 (**G**,**H**) and α5-α3 (**I**,**J**) interfaces of α3β4α5 nAChRs (2:2:1 stoichiometry). The ligands are displayed as balls and sticks, the NIC-binding residues are shown as sticks (the residues with side chains colored in blue and marked with “+” belong to the principal side while the residues with side chains colored in green and marked with “–” belong to the complementary side). The hydrogen bonds are shown as green dashed lines and the rest of the receptor as molecular surface.

**Figure 2:**
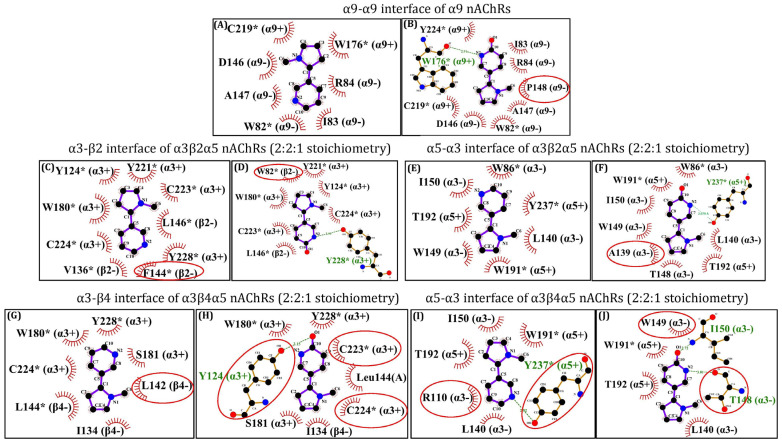
Two-dimensional representation of the interactions between (*S*)-nicotine or (*S*)-6-hydroxynicotine and the NIC-binding residues of the α9-α9 interface of α9 nAChRs (**A**,**B**), α3-β2 (**C**,**D**) and α5-α3 (**E**,**F**) interfaces of α3β2α5 nAChRs (2:2:1 stoichiometry) and α3-β4 (**G**,**H**) and α5-α3 (**I**,**J**) interfaces of α3β4α5 nAChRs (2:2:1 stoichiometry). The residues marked with * are identified in the literature as interacting with the ligand, while the encircled residues are new residues putatively interacting with the ligand. The hydrogen bonds and the hydrophobic interactions between the residues and the corresponding atoms of the ligands are displayed according to Ligplot^+^ software v2.2. The residues marked with “+” and “–” belong to the principal and complementary sides, respectively.

Next, (*S*)-nicotine and (*S*)-6-hydroxynicotine were docked to the α9-α9 interface of the α9 nAChR, α3-β2 and α5-α3 interfaces of α3β2α5 nAChRs (2:2:1 stoichiometry) and α3-β4 and α5-α3 interfaces of α3β4α5 nAChRs (2:2:1 stoichiometry). The theoretical binding energies, ligand efficiencies, and the number of hydrogen (H) bonds formed with the receptor were calculated for the best three binding positions of each ligand (Supplementary Table S3). As depicted in Figure 1A,B, for NIC and 6HLN a rather similar orientation in the binding pocket located at the α9-α9 interface of the homopentameric α9 nAChR was noticed. Additionally, 6HLN formed an extra H bond with a tryptophan residue (W176) found on the principal side (α9+) of the α9-α9 interface and exhibited a lower binding energy compared to NIC (−7.38 vs. −7.12), thus suggesting an increased affinity of 6HLN towards this receptor. In addition to the residues described in the literature, Ligplot^+^ software v2.2 suggests that the ligands could also interact with I83, R84, D146, and A147 residues, and particularly P148 for 6HLN, from the complementary side (α9-) of the α9-α9 interface (Figure 2A,B).

For the heteropentameric α3β2α5 and α3β4α5 subtypes of nAChRs, the docking has been performed only at the α3-β2/β4 and α5-α3 interfaces, based on the fact that NIC was experimentally found only at α-α and α-β interfaces. In the α3β2α5 nAChRs, a very similar orientation has been identified between NIC and 6HLN at the α3-β2 (Figure 1C,D) and α5-α3 (Figure 1E,F.) interfaces, along with a lower binding energy for the 6HLN compared to the NIC (−6.69 vs. −6.48 and −6.28 vs. −5.71 respectively). Additionally, ADT (AutoDockTools) identified an extra H bond formed between 6HLN and the Y228 residue located on the principal side of the α3-β2 interface (Figure 1D) and with the Y237 residue located on the principal side of the α5-α3 interface (Figure 1F).

At the α3-β2 interface (Figure 2C,D), Ligplot^+^ software v2.2 revealed that the ligands are interacting with the residues described in the literature [23]. However, at the α5-α3 interface, several new residues, such as T192 on the principal side (α5+) and A139, K138, L140, T148, W149, and I150 on the complementary side (α3-), could be involved in the interaction with the ligands (Figure 2E,F). A similar orientation of the ligands was also observed at the α3-β4 (Figure 1G,H) and α5-α3 (Figure 1I,J) interfaces of α3β4α5 nAChRs. Consistent with previous docking simulations, compared to NIC, a lower binding energy of 6HLN in the α3-β4 (−6.55 vs. −6.07) and α5-α3 (−6.05 vs. −5.82) binding sites was noticed, suggesting a higher affinity for the NIC derivative. However, ADT did not reveal any H bonds between 6HLN and the residues of the α3-β4 binding site but revealed one H bond formed between NIC and Y237 on the principal side (α5+) and two H bonds formed between 6HLN and T148 and I150 residues located on the complementary side (α3-) of the α5-α3 interface. Ligplot^+^ software v2.2 showed a potential H bond formed between 6HLN, but not NIC, and the Y124 residue of the α3-β4 interface (Figure 2G,H) and supports the ADT-formed H bonds between NIC and 6HLN with the Y237 and I150 and T148 residues, respectively, of the α5-α3 interface (Figure 2I,J). Collectively, these results suggest that 6HLN might bind to the homopentameric α9 and heteropentameric α3β2α5 and α3β4α5 subtypes of nAChRs with higher affinity than NIC due to the extra H bonds formed between 6HLN and the residues of the binding sites.

Although in silico data indicate the subtypes of nAChRs that interact with 6HLN, we lack information regarding the expression of these subtypes in cancer cells. For the next investigations, we have selected representative and highly used cell lines for some cancer types.

### 2.2. Influence of 6HLN and NIC on Proliferative Behavior

**Figure 3:**
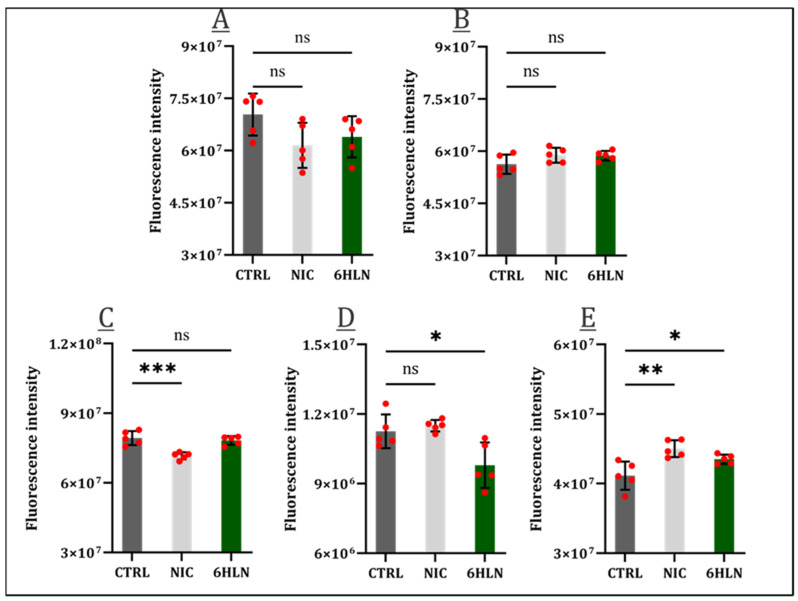
Cellular viability 48 h post incubation of NIC/6HLN (50 nM) in (**A**) 16HBE14o, (**B**) MCF10A, (**C**) A549, (**D**) MCF7, and (**E**) U87 cell lines. Values are means ± S.D. (n = 5). (**C**) CTRL vs. NIC: *** *p* = 0.0003. (**D**) CTRL vs. 6HLN: * *p* = 0.0138. (**E**) CTRL vs. NIC: ** *p* = 0.0018; CTRL vs. 6HLN: * *p* = 0.0386. ns—non-significant.

6HLN’s effects on the cellular viability of both normal (Supplementary Figure S5) and cancer (Supplementary Figure S6) cell lines have been assessed. As the 16HBE14o normal bronchial cell line showed sensitivity to a concentration equal to or higher than 100nM for 6HLN (Supplementary Figure S5) or NIC [24], the reference nAChRs agonist, a concentration of 50 nM 6HLN, was further used in this study. When administered, 6HLN did not elicit detrimental effects in either the 16HBE14o (Figure 3A) normal bronchial cell line or in the MCF10A (Figure 3B) normal breast cell line. The administration of NIC at a concentration of 50 nM similarly did not influence the viability of the 16HBE14o (Figure 3A) or MCF10A (Figure 3B). As opposed to normal cell lines, all tumor cell lines reacted differently in response to the administration of either 6HLN or NIC. So, 6HLN did not show any influence regarding the viability of A549 cells (Figure 3C), while NIC induced a significant viability decrease.

On the contrary, in the MCF7 breast cell line, 6HLN significantly reduced the cells’ viability, while NIC had neutral effects (Figure 3D). Only in the U87 glioblastoma cell line 6HLN and NIC induced similar effects, both compounds significantly increasing cell viability (Figure 3E).

**Figure 4:**
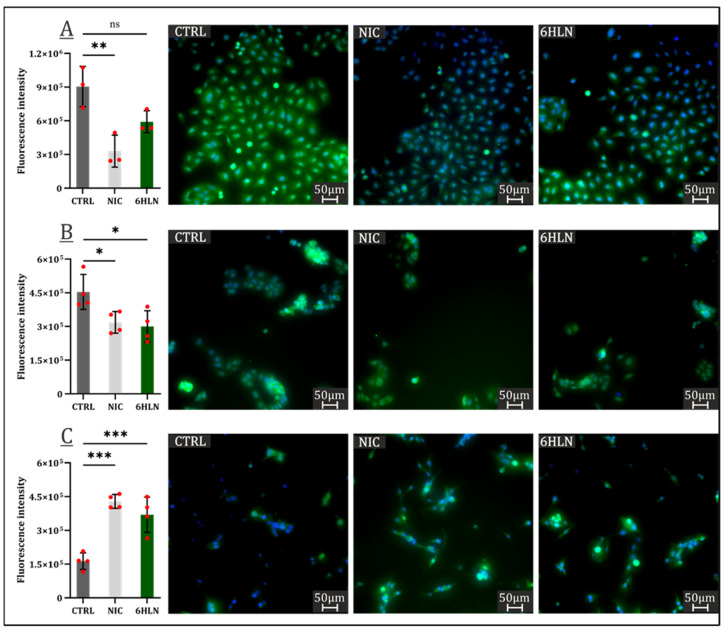
AKT3 expression 48 h post incubation of NIC/6HLN (50 nM) in (**A**) A549, (**B**) MCF7, and (**C**) U87 cell lines. Values are means ± S.D. (n = at least 3). (**A**) CTRL vs. NIC: ** *p* = 0.0049. (**B**) CTRL vs. NIC: * *p* = 0.0333; CTRL vs. 6HLN: * *p* = 0.0178. (**C**) CTRL vs. NIC: *** *p* = 0.0001; CTRL vs. 6HLN: *** *p* = 0.0007. ns—non-significant.

One of the most frequently dysregulated pathways in human malignancies is PI3KAkt/mTOR, downstream effectors of this pathway being aberrantly expressed in 50% of tumors, which translates into enhanced cell survival and proliferation [25]. AKT overexpression is frequently indicative of an overall poor prognosis, despite being proven that the AKT isoforms (AKT1, AKT2, and AKT3) present distinct and often opposing roles within tumorigenesis [26]. Hence, the AKT3 expression upon 6HLN and NIC administration has been evaluated, and, at least in the case of lung cancer cells, 6HLN did not induce a significant variation of AKT3 expression, with only a modest downregulation of this protein expression being detected (Figure 4A). However, in both mammary and brain cancers, 6HLN induced significant, yet opposing effects, downregulating the expression of AKT3 in MCF7 cells (Figure 4B), while upregulating it in U87 cells (Figure 4C). It can be noticed that the effects of 6HLN on AKT3 expression stand in perfect agreement with those observed when investigating the viability levels in response to 6HLN administration in cancerous cell lines. Regarding NIC influence, significant downregulations of AKT3 expression were detected in A549 (Figure 4A) and MCF7 cells (Figure 4B), while a significant AKT3 upregulation was observed in U87 cells (Figure 4C). The effects of NIC on AKT3 expressions were positively correlated with the impact of NIC on cancerous cell lines viability, the exception being represented by the MCF7 cell line, where the downregulated AKT3 expression does not translate in a decreased viability level.

AKT3 upregulation is associated with the biogenesis of various malignancies, inducing detrimental or beneficial effects depending on the type of cancer. So, AKT3 upregulation is associated with a poor prognosis for breast cancer patients, but a good prognosis for glioblastoma patients [27,28]. In this study, 6HNL increased the AKT3 level in U87 cells in a significant manner, but it also increased cellular viability; further studies are needed for a complete picture of 6HNL influence in glioblastoma cells. Regarding A549 lung adenocarcinoma, 6HLN appears to not interfere with the behavior of these cells, with both viability and AKT3 levels remaining relatively constant. In MCF7 cells, significant decreases in both viability level and AKT3 expression were detected upon 6HLN administration, suggesting that additional assessments would be necessary to fully understand its influence on breast cancer cells.

### 2.3. Influence of 6HLN and NIC on Migratory Behavior

**Figure 5:**
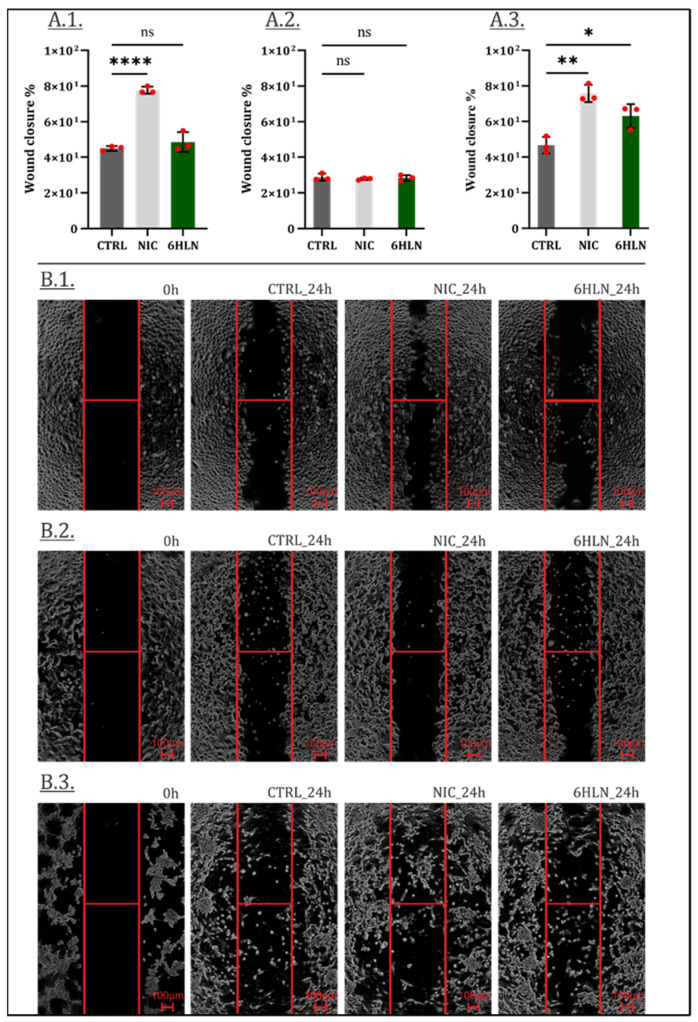
Wound closure 24 h post incubation of NIC/6HLN (50 nM) in (**A.1**) A549, (**A.2**) MCF7 and, (**A.3**) U87 cells. B—representative pictures: (**B.1**) A549, (**B.2**) MCF7 and (**B.3**) U87 cells. Values are means ± S.D. (n = 3). (**A.1**) CTRL vs. NIC: **** *p* < 0.0001. (**A.3**) CTRL vs. NIC: ** *p* = 0.0012; CTRL vs. 6HLN: * *p* = 0.0188. ns—non-significant.

**Figure 6:**
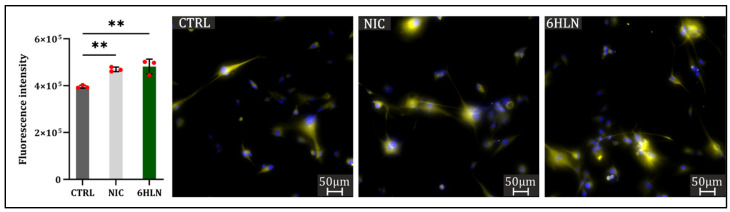
Vimentin expression 48 h post incubation of NIC/6HLN (50 nM) in U87 cell line. Values are means ± S.D. (n = 3). CTRL vs. NIC: ** *p* = 0.0060; CTRL vs. 6HLN: ** *p* = 0.0028.

Previous studies showed that nAChR activation may induce structural changes and cytoskeleton remodeling, influencing cellular motility [29,30]. Therefore, following 6HLN administration, the migratory behavior of both normal (Supplementary Figure S7) and cancer cell lines (Figure 5) was investigated. Following previous reports [31,32], this study showed that NIC-stimulated A549 and NIC-stimulated U87 cells covered the wound area at a faster rate than the unstimulated cells (Figure 5(A.1,A.3)), while no differences were noted between NIC-stimulated and NIC-unstimulated MCF7 cells (Figure 5(A.2). More importantly, 6HLN appeared to induce little to no changes in the motility rate of both A549 or MCF7 cells (Figure 5(A.1,A.2)), presumably not sustaining the metastatic potential of these cells. However, an increased motility rate has been detected in 6HLN-stimulated U87 cells (Figure 5(A.3)), which has been further correlated with an increased vimentin expression (Figure 6), indicative of epithelial to mesenchymal transition (EMT) program activation [33].

In addition to classical hallmarks, including downregulated expressions of E-cadherin, zona occludens 1 (ZO-1), occludin and cytokeratin, and upregulated expressions of fibronectin, α-smooth muscle actin (α-SMA), fibroblast-specific protein 1 (FSP-1), N-cadherin, and vimentin, EMT also involves the expression of proteases, including matrix metalloproteases (MMPs) [34].

**Figure 7:**
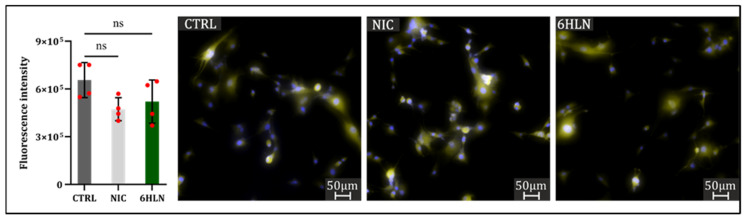
MMP9 expression 48 h post incubation of NIC/6HLN (50 nM) in U87 cell line. Values are means ± S.D. (n = 4). ns—non-significant.

However, in this study, increased U87 cell motility, correlated with vimentin upregulation, has not been positively associated with MMP9 expression, but rather a modest downregulation of MMP9 has been induced by both 6HLN and NIC (Figure 7).

Such inconsistencies between increased motility rate and unchanged MMP9 expression have been previously reported for NIC, McConnell et al. [35] show that MMP9 expression remained constant even when glioblastoma cells were stimulated with 500 nM NIC. As the MMP9 is not the only matrix metalloprotease involved in glioblastoma [36], other MMPs may mediate the increased motility observed in U87 cells. These pro-migratory effects correlated with the increased cellular viability revealed that in glioblastoma cells, 6HLN follows similar pathways as NIC, mainly determining identical side effects.

### 2.4. Influence of 6HLN and NIC on Pro-Inflammatory Cytokines

Considering that inflammatory responses have been correlated with all stages of malignant progression [37] and that anti-inflammatory drugs have been associated with a reduced incidence of a wide range of cancers [38], the usage of anti-inflammatory drugs has been perceived as a logical approach in the fields of chemoprevention and cancer treatment [39]. Nicotine has been described as a potent anti-inflammatory alkaloid, being regarded as a proper therapeutic approach in a variety of inflammatory diseases due to its ability to regulate various immune factors, including tumor necrosis factor α (TNFα), interleukin 1α (IL1α), interleukin 1β (IL1β), interleukin 4 (IL4), interleukin 6 (IL6), granulocyte–macrophage colony-stimulating factor (GM-CSF) cytokines, and β-defensin [40]. Anti-inflammatory properties of 6HLN have also been proven in vivo Alzheimer’s disease models, efficiently reducing β-amyloid-induced IL1β expression [16].

**Figure 8:**
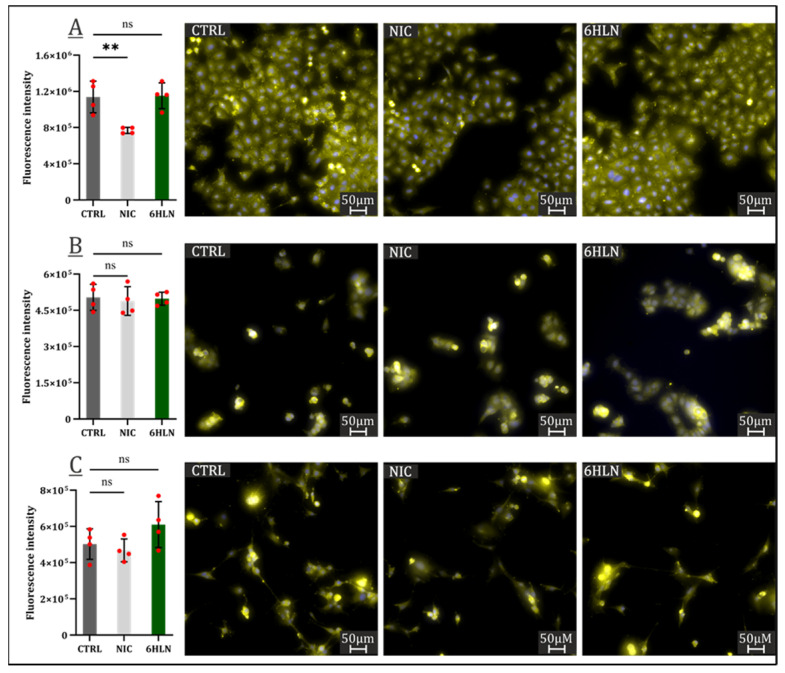
IL6 expression 48 h post incubation of NIC/6HLN (50 nM) in (**A**) A549, (**B**) MCF7, and (**C**) U87 cell lines. Values are means ± S.D. (n = 4). (A) CTRL vs. NIC: ** *p* = 0.0060. ns—non-significant.

IL1β and IL6 upregulated expressions are seen as almost universal predictors of poor patient outcomes in cancer [41,42,43,44]. This study indicated a minimal impact of 6HLN and NIC on IL6 expression (Figure 8), with NIC significantly reducing IL6 expression only in A549 cells (Figure 8A).

**Figure 9:**
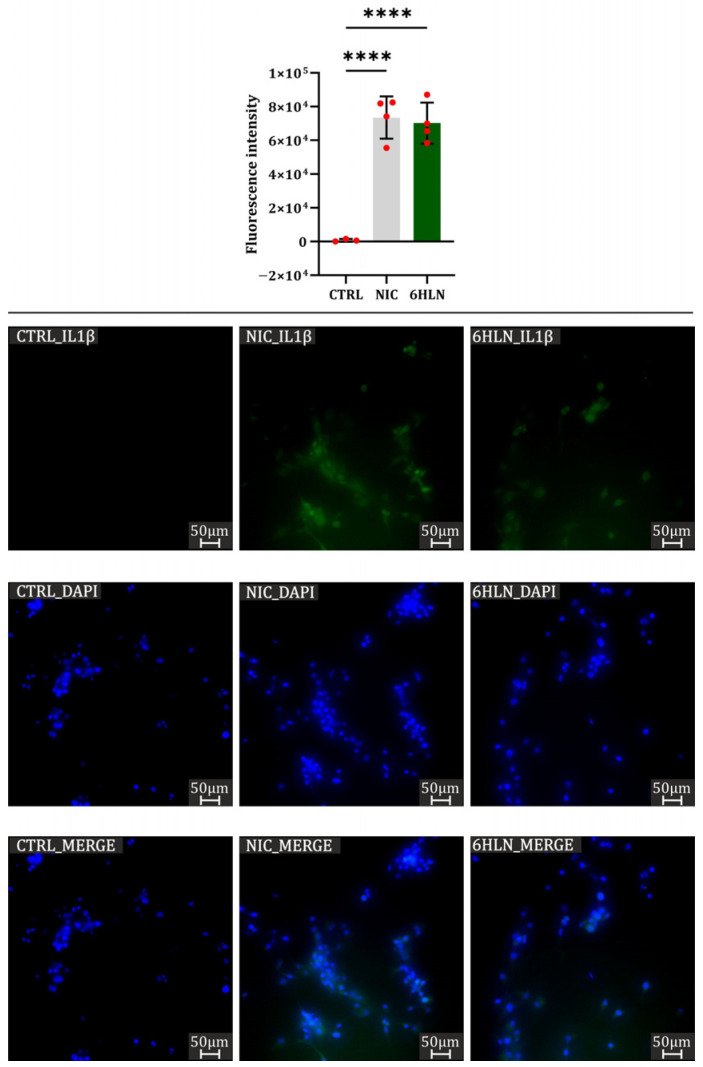
IL1β expression 48 h post incubation of NIC/6HLN (50 nM) in U87 cell line. Values are means ± S.D. (n = at least 3). CTRL vs. NIC: **** *p* < 0.0001; CTRL vs. 6HLN: **** *p* < 0.0001.

However, in the U87 cell line, both 6HLN and NIC induced massive expression of IL1β pro-inflammatory cytokine (Figure 9). In A549 and MCF7 cell lines, IL1β expression has not been detected using the immunofluorescence technique as a consequence of extremely low IL1β expression in the absence of deleterious stimuli [45].

Hence, it is assumed that neither NIC nor 6HLN is an inducer of IL1β in A549 and MCF7 cells, but this fact should be confirmed via more sensitive techniques in cytokines’ quantification.

## 3. Materials and Methods

### 3.1. Cell Cultures

16HBE14o (human normal bronchial epithelial cell line (SCC150, Sigma—Aldrich, Darmstadt, Germany)) cells were grown in airway epithelial cell basal medium (PCS-300-030, American Type Culture Collection, Manassas, VA, USA) supplemented with a bronchial epithelial cell growth kit (PCS-300-040, American Type Culture Collection, Manassas, VA, USA), while MCF10A (human normal breast epithelial cell line (CRL-10317, American Type Culture Collection, Manassas, VA, USA)) cells were grown DMEM: F-12 medium (30-2006, American Type Culture Collection, Manassas, VA, USA) supplemented with 10% horse serum (H1270, Sigma—Aldrich, Darmstadt, Germany), 10 ng/mL cholera toxin (C8052, Sigma—Aldrich, Darmstadt, Germany), 0.5 ng/mL hydrocortisone (H0888, Sigma—Aldrich, Darmstadt, Germany), 10 ug/mL insulin (I1882, Sigma—Aldrich, Darmstadt, Germany), 20 ng/mL epidermal growth factor (E9644, Sigma—Aldrich, Darmstadt, Germany), and 1% penicillin–streptomycin (03-031-1B, Biological Industries, Beit HaEmek, Israel). The tumoral A549 (human epithelial lung carcinoma cell line (CRM-CCL-185, American Type Culture Collection, Manassas, VA, USA) cells were cultured in RPMI—1640 medium (01-106-1A, Biological Industries, Beit HaEmek, Israel), MCF7 human epithelial breast adenocarcinoma cell line (HTB-22, American Type Culture Collection, Manassas, VA, USA) cells were cultured in DMEM: F-12 medium, and U87 human epithelial glioblastoma cell line (HTB-14, American Type Culture Collection, Manassas, VA, USA)—generously gifted by James Lorens (BerGen Bio AS, Bergen, Norway)—cells were cultured in DMEM (30-2002, American Type Culture Collection, Manassas, VA, USA), all three media being supplemented with 10% fetal bovine serum (F7524, Sigma—Aldrich, Darmstadt, Germany) and 1% penicillin–streptomycin. All cell lines were kept at 37 °C in a 5% CO_2_ atmosphere. Unless mentioned otherwise, upon reaching confluency, the cells were seeded in a 96-well flat-bottom tissue culture plates at different densities, as follows: 5000 cells/well for 16HBE14o and MCF10A cell lines, 3500 cells/well for MCF7 cell line, 2500 cells/well for U87 cell line, and 2000 cells/well for A549 cell line. Twenty-four hours post-seeding, the cells were incubated with 6-Hydroxy-L-nicotine (6HLN) (SC-394077, Santa-Cruz Biotechnology, Dallas, TX, USA)/nicotine (NIC) (N3876, Sigma—Aldrich, Darmstadt, Germany) in a concentration of 50 nM for 48 h.

### 3.2. Cell Viability

Cellular viability was monitored by CellTiter-Blue^®^ Cell Viability assay (G8081, Promega, Madison, WI, USA), following the manufacturer’s instructions. Hence, 48 h after NIC/6HLN administration, 20 µL of reagent containing resazurin was added to each well and the cells were allowed to convert resazurin to resorufin for three hours at 37 °C under 5% CO_2_. The resulting fluorescence was recorded using FilterMax F5 (Molecular Devices, San Jose, CA, USA) microplate reader.

### 3.3. Wound Healing Assay

A Culture-Insert 2 Well in μ-Dish ^35 mm^ (80206, Ibidi, Gräfelfing, Germany) was used to assess cell motility following the manufacturer’s specifications. Different concentrations of cell suspensions were prepared depending on the growth rate of each cell line and 70 μL of each cell suspension was seeded into each well of the Culture-Insert. All cells were allowed to grow at 37 °C under 5% CO_2_ until a confluent cell layer had been obtained, then the cells were subjected to serum starvation for 24 h. Upon the removal of cell inserts, the cell layers were washed with PBS (02-023-1A, Biological Industries, Beit HaEmek, Israel) and a volume of 2 mL of growth media supplemented with 0.5% fetal bovine serum and 50 nM NIC/6HLN was added to each μ-Dish. Pictures of the gaps were acquired at 4X immediately (0 h) and 24 h post incubation at 37 °C under 5% CO_2_ using a Nikon Eclipse TS2R microscope with Mshot Digital Imaging Software (V1.1.6). The acquired pictures were analyzed using the wound healing size plugin for ImageJ and the wound closure was determined by the following formula: ((A_t = 0_ − A_t = Δt_)/A_t = 0_) × 100, where A_t = 0_ represents the initial wound area and A_t = Δt_ represent the wound area 24 h post initial scratch [46].

### 3.4. Immunocytofluorescence

A549, MCF7, and U87 cells were exposed to 50 nM NIC/6HLN for 48 h and their corresponding controls were fixed with 4% paraformaldehyde (A11313, Thermo Scientific Chemicals, Waltham, MA, USA), washed with PBS to remove traces of fixation solution, and permeabilized using a permeabilization mix of 0.3% digitonin (G9441, Promega, Madison, WI, USA), 0.3% Triton X 100 (X100, Sigma—Aldrich, Darmstadt, Germany), and 0.3% saponin (558255, Merck, Darmstadt, Germany). The cells were incubated with anti-AKT3 (E1Z3W, Cell Signaling Technology, Danvers, MA, USA), anti-MMP9 (SC-21733, Santa-Cruz Biotechnology, Dallas, TX, USA), anti-vimentin (M0725, Dako REAL, Santa Clara, CA, USA), anti-IL-1β (D3U3E, Cell Signaling Technology, Danvers, MA, USA), and anti-IL6 primary antibodies (SC-130326, Santa-Cruz Biotechnology, Dallas, TX, USA), diluted into antibody diluent (S202230-2, Dako REAL, Glostrup, Denmark) (1:25), and incubated at 4 °C for 72 h. Following two washing steps with PBS, the cells were further incubated with Alexa Fluor 546 Goat anti-Mouse IgG (A-11030, ThermoFisher Scientific, Waltham, MA, USA) or fluorescein goat anti-rabbit IgG (F2765, ThermoFisher Scientific, Waltham, MA, USA) secondary antibodies (diluted 1:200 into antibody diluent) at 4 °C for 24 h. Then, the cells were washed twice with PBS to remove the unbound secondary antibodies and incubated overnight with Prolong Gold antifade (P36935, ThermoFisher Scientific, Waltham, MA, USA) with DAPI at 4°C. All pictures were acquired at 20× magnification using a Zeiss Axio Observer Z1 Microscope (Hamamatsu C11440-22C camera) from TissueGnostic rig and TissueFAXS 6.146 software. The sum intensity of the fluorescence signal corresponding to each event was quantified through TissueQuest 6.0.1.126 (Vienna, Austria) software.

### 3.5. AlphaFold2 Multimer Structure Prediction of α9, α3β2α5 and α3β4α5 nAChRs and Molecular Docking Simulations

The tridimensional (3D) structures of the homopentameric α9 nAChRs and heteropentameric α3β2α5 (2:2:1 stoichiometry) and α3β4α5 (2:2:1 stoichiometry) nAChRs were predicted from the corresponding FASTA sequences of the human α9 subunit (Genbank accession no. NP_060051.2), α3 subunit (Genbank accession no. NP_000743.5), α5 subunit (Genbank accession no. NP_000745.4), β2 subunit (Genbank accession no. NP_000748.3), and β4 subunit (Genbank accession no. NP_000750.5) using AlphaFold 2 multimer [47] with default parameters. Post prediction, 25 structures were generated and ranked based on their predicted Local Distance Difference Test (pLDDT) confidence score. The highest-ranked conformation was chosen and visualized with UCSF ChimeraX v1.7.1 [48]. To identify the NIC-binding residues in the predicted structures, the protein sequences of α9, α3, α5, β2, and β4 subunits were aligned with those of α4 and β2 subunits of α4β2 subtype of nAChRs (3α:2β stoichiometry, PDB ID 6CNK). For in silico docking experiments, the highest-ranking predicted structures of α9, α3β2α5, and α3β4α5 subtypes of nAChRs were used as a receptor. The structures were edited in UCSF ChimeraX so that only two subunits (one interface) forming an NIC-binding site remained. The 3D structures of the ligands, (*S*)-nicotine (CID 89594) and (*S*)-6hydroxynicotine (CID 439383), were downloaded from the PubChem database as sdf and converted to a suitable format for docking using Frog v2.14—free online drug conformation generation [49]. In silico molecular docking was performed using the genetic algorithm of AutoDock 4 [50] and the molecular surface generation, energy grid, and search box were performed with AutoDockTools v1.5.7 [51] with all parameters on default. The structure of the receptor was kept rigid, and the ligands were flexible, with all bonds rotatable. Targeted docking was performed with a search area defined as a box of 60 × 50 × 50 Å, centered on the NIC-binding pocket. Following molecular docking simulations, 50 conformations were obtained for each ligand and ranked based on the calculated theoretical binding energies. RMSD was calculated for the best three binding poses of (s)-nicotine and (s)-6-hydroxynicotine using UCSF ChimeraX [52]. Bidimensional (2D) diagrams of the complex were generated using LigPlot^+^ v2.2 [53] and 3D visualized using ChimeraX.

### 3.6. Statistical Analysis

All statistical analysis was performed using GraphPad Prism v9.1.0 software (La Jolla, CA, USA) by applying the one-way analysis of variance (ANOVA), followed by Dunnet’s post hoc test. Data analysis results are expressed as the mean ± standard deviation (S.D.). A value of *p*  <  0.05 was set for statistical significance.

## 4. Conclusions

To the authors’ knowledge, this is the first study evaluating 6HLN effects in cancer. 6HLN appears to induce distinct, even opposing effects, depending on the cancer type. Thus, A549 lung cancer cells appear unaffected by 6HLN administration, with no changes in cellular viability, migratory behavior, or inflammatory status detected.

However, in U87 glioblastoma cells, 6HLN mainly exhibited cancer-stimulatory effects by increasing cell viability, enhancing migratory properties supported by vimentin overexpression, and upregulating the expression of the IL1β proinflammatory protein.

Alternatively, in MCF7 breast cancer cells, 6HLN administration did not affect migratory abilities or proinflammatory marker expression but reduced cellular viability, indicating possible cancer-inhibitory effects. In silico data suggest that 6HLN might exert its effects by binding to nAChRs. Therefore, further studies would be of interest for a better understanding of cholinergic neurotransmission’s role in cancer pathologies, as variations in nAChRs’ conformation and abundance across cell lines may lead to distinct specific or nonspecific cellular responses.

## Data Availability

The original contributions presented in the study are included in the article/Supplementary Material; further inquiries can be directed to the corresponding authors.

## Supplementary Materials

The following supporting information can be downloaded at: https://www.mdpi.com/article/10.3390/molecules29235593/s1, Figure S1: The AlphaFold multimer-modeled structure of homopentameric α9 nAChRs, the NIC-binding residues at the α9-α9 interface and the confidence level expressed as pLDDT score; Figure S2: The AlphaFold multimer-modeled structure of heteropentameric α3β2α5 (2:2:1 stoichiometry) nAChRs, the NIC-binding residues at the α5-α3 and α3-β2 interfaces and the confidence level expressed as pLDDT score; Figure S3: The AlphaFold multimer-modeled structure of heteropentameric α3β4α5 (2:2:1 stoichiometry) nAChRs, the NIC-binding residues at the α5-α3 and α3-β4 interfaces and the confidence level expressed as pLDDT score; Figure S4: The native position of NIC molecule (**A**,**C**) and the best theoretical binding pose of (S)-NIC (**B**,**D**) in the α4-α4 interface (top panel) and α4-β2 interface (bottom panel) of α4β2 nAChRs (3α:2β stoichiometry); Figure S5: Cellular viability 24 and 48 h post incubation of 6HLN at different concentrations (10nM, 50 nM, 100 nM, and 1 µM) in (**A**) 16HBE14o and (B) MCF10A normal cell lines. Values are means ± S.D. (n = 5). ns—non-significant, * *p* ≤ 0.05; *** *p* ≤ 0.001; Cellular viability 24 and 48 h post incubation of 6HLN at different concentrations (10 nM, 50 nM, 100 nM, and 1 µM) in (A) A549, (B) MCF-7, and (C) U87 cancer cell lines. Values are means ± S.D. (n = 5). ns—non-significant, * *p* ≤ 0.05; ** *p* ≤ 0.01; Figure S6: Cellular viability 24 and 48 h post incubation of 6HLN at different concentrations (10 nM, 50 nM, 100 nM, and 1 µM) in (A) A549, (B) MCF-7, and (C) U87 cancer cell lines. Values are means ± S.D. (n = 5). ns—non-significant, * *p* ≤ 0.05; ** *p* ≤ 0.01; Figure S7: Wound closure 24 h post incubation of NIC/6HLN (50 nM) in 16HBE14o (A1) and MCF10A (A2) cells. B—representative pictures: 16HBE14o (B1) and MCF10A (B2) cells. Values are means ± S.D. (n = 3); Table S1: The correspondence between the nicotine-binding residues of α4 subunit of α4β2 nAChRs and α9, α3 and α5 subunits of α9, α3β2α5, and α3β4α5 subtypes of nAChRs; Table S2: The correspondence between the nicotine-binding residues of α4 subunit of α4β2 nAChRs and α9, α3, and α5 subunits of α9, α3β2α5, and α3β4α5 subtypes of nAChRs; Table S3: The binding energies, ligand efficiencies, and the number of hydrogen (H) bonds formed for the best three binding positions of the ligands in the corresponding receptor.
