# Supplementary material for: The Distinct Biological Effects of 6-Hydroxy-L-Nicotine in Representative Cancer Cell Lines
Source: Molecules. 2024 Nov 26;29(23):5593. doi: 10.3390/molecules29235593 (PMC11643659; doi:10.3390/molecules29235593)
Supplement: Supplementary file 1 [file molecules-29-05593-s001.zip › molecules-3297934-supplementary.pdf]

Supplementary material

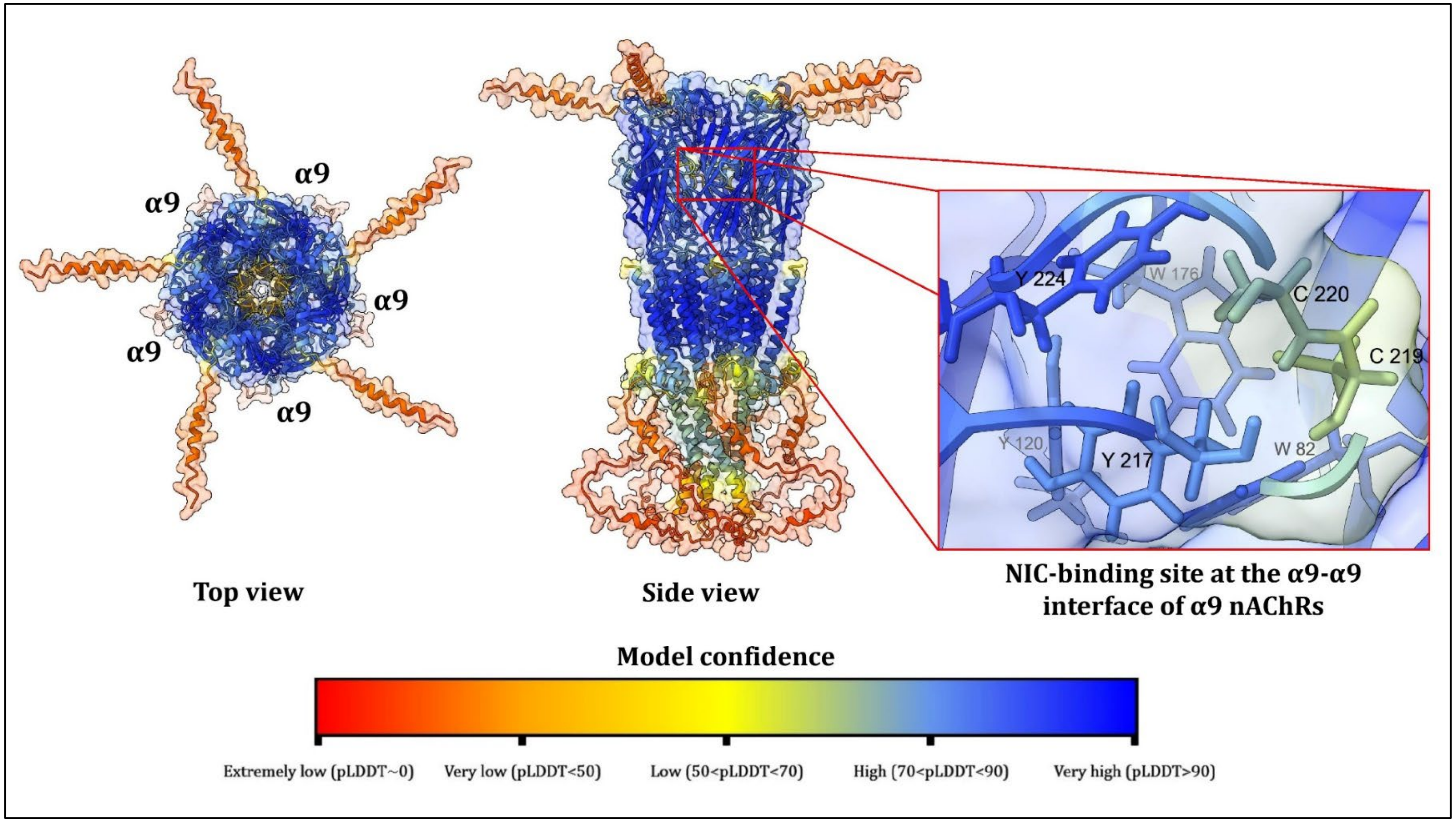

Supplementary Figure S1. The Alphafold multimer-modeled structure of homopentameric α9 nAChRs, the NIC-binding residues at the α9-α9 interface and the confidence level expressed as pLDDT score.

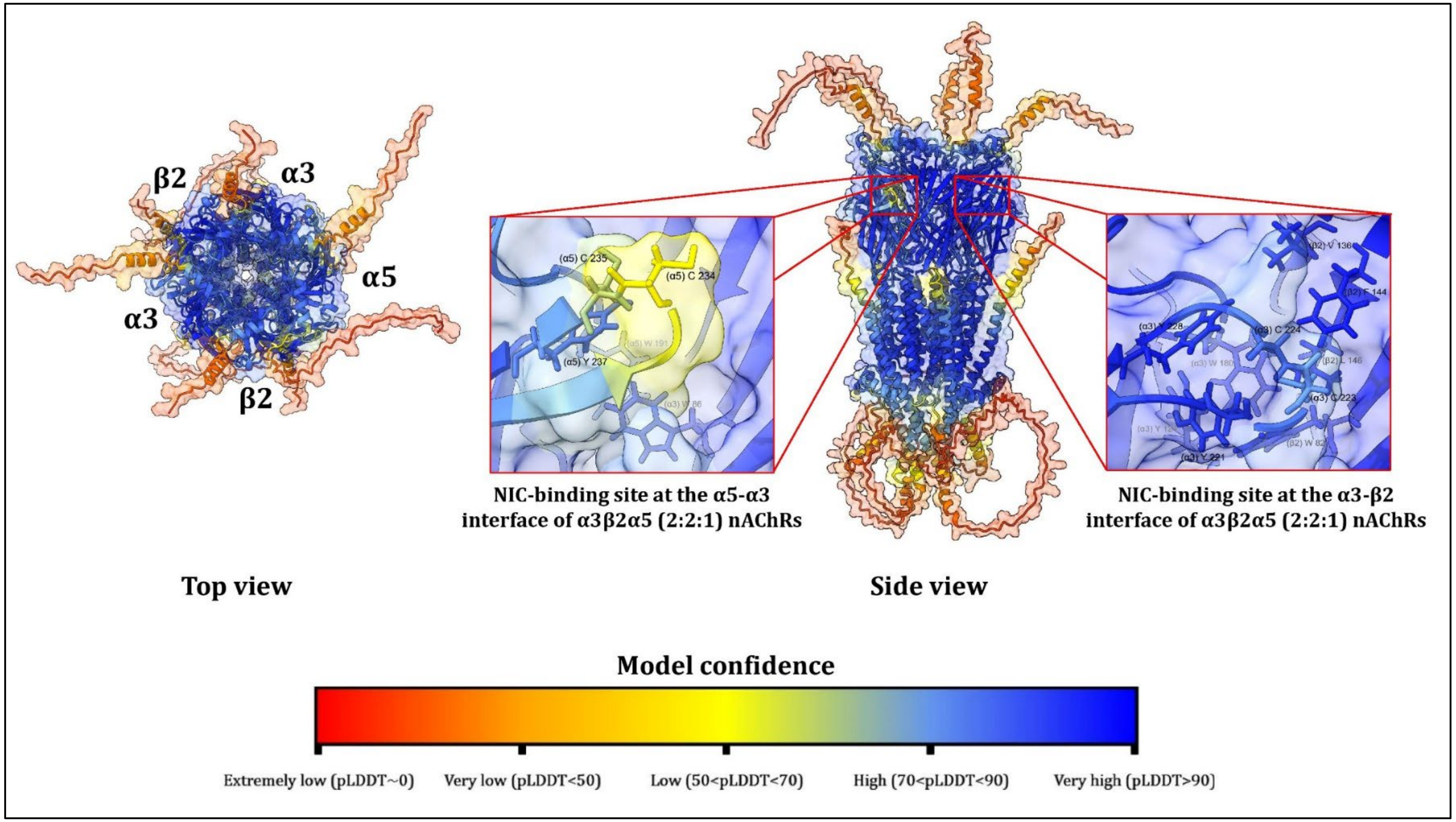

Supplementary Figure S2. The Alphafold multimer-modeled structure of heteropentameric α3β2α5 (2:2:1 stoichiometry) nAChRs, the NIC-binding residues at the α5-α3 and α3-β2 interfaces and the confidence level expressed as pLDDT score.

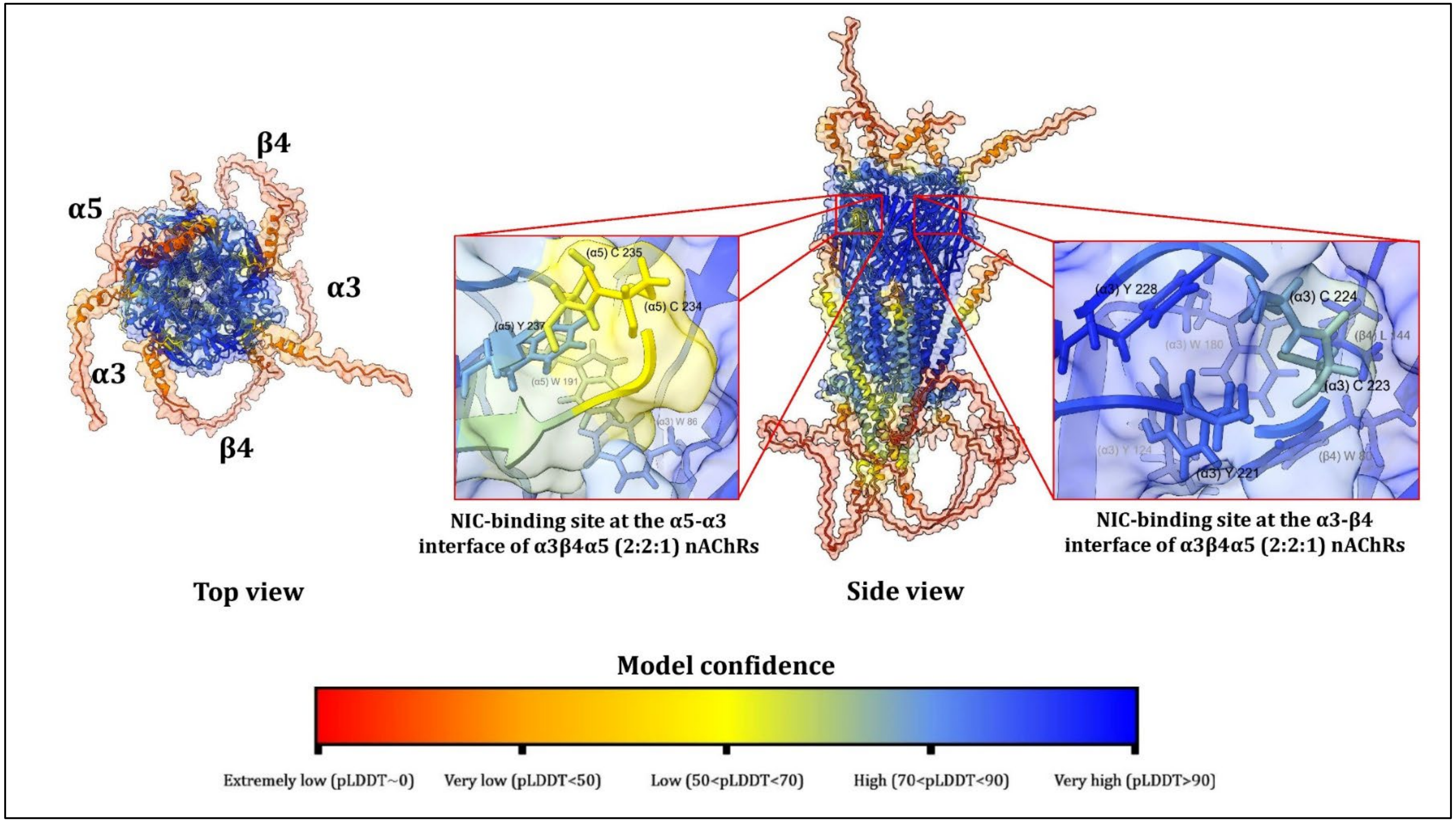

Supplementary Figure S3. The Alphafold multimer-modeled structure of heteropentameric α3β4α5 (2:2:1 stoichiometry) nAChRs, the NIC-binding residues at the α5-α3 and α3-β4 interfaces and the confidence level expressed as pLDDT score.

Supplementary Table S1. The correspondence between the nicotine-binding residues of α4 subunit of α4β2 nAChRs and α9, α3 and α5 subunits of α9, α3β2α5 and α3β4α5 subtypes of nAChRs

		**NIC-binding site residues**	**Equivalent residues for NIC binding site in the predicted structures***				
		**α4β2 nAChRs (3α:2β, PDB ID 6CNK)**	**α9 nAChRs**	**α3β2α5 (2:2:1) nAChRs**		**α3β4α5 (2:2:1) nAChRs**	
		**α4 subunit**	**α9 subunit**	**α3 subunit**	**α5 subunit**	**α3 subunit**	**α5 subunit**
**α subunit**	**Principal side**	Y100	Y120 (90.6)	Y124 (95.1)	-	Y124 (93.9)	-
		W156	W176 (92.6)	W180 (97)	W191 (92.2)	W180 (95.3)	W191 (82.3)
		Y197	Y217 (89.3)	Y221 (95.7)	-	Y221 (93.1)	-
		C199	C219 (79.6)	C223 (90)	C234 (66.5)	C223 (84.9)	C234 (63.8)
		C200	C220 (83.2)	C224 (92.8)	C235 (81.7)	C224 (88)	C235 (75.6)
		Y204	Y224 (95.3)	Y228 (97.9)	Y237 (93)	Y228 (97.2)	Y237 (89.4)
	**Complementary side**	W62	W82 (83.8)	W86 (96.3)	W98 (95.5)	W86 (95.9)	W98 (94.7)
		H116	-	-	-	-	-
		Q124	-	-	-	-	-
		T126	-	-	T161 (95.4)	-	T161 (94.9)

*The predicted Local Distance Difference Test (pLDDT) scores for the residues are indicated in brackets.

Supplementary Table S2. The correspondence between the nicotine-binding residues of α4 subunit of α4β2 nAChRs and α9, α3 and α5 subunits of α9, α3β2α5 and α3β4α5 subtypes of nAChRs

		NIC-binding site residues	Equivalent residues for NIC-binding site in the predicted structures*	
		**α4β2 nAChRs (3α:2β, PDB ID 6CNK)**	**α3β2α5 nAChRs (2:2:1)**	**α3β4α5 nAChRs (2:2:1)**
		**β2 subunit**	**β2 subunit**	**β4 subunit**
**β subunit**	**Principal side**	Y95	Y120 (94.7)	Y118 (93.6)
		R149	R174 (96.7)	R172 (95.4)
		W151	W176 (93.5)	W174 (94)
		Y196	Y221 (95.7)	Y219 (93.8)
	**Complementary side**	W57	W82 (97.7)	W80 (96.7)
		V111	V136 (98)	-
		F119	F144 (98.2)	-
		L121	L146 (98)	L144 (96.8)

*The predicted Local Distance Difference Test (pLDDT) scores for the residues are indicated in brackets.

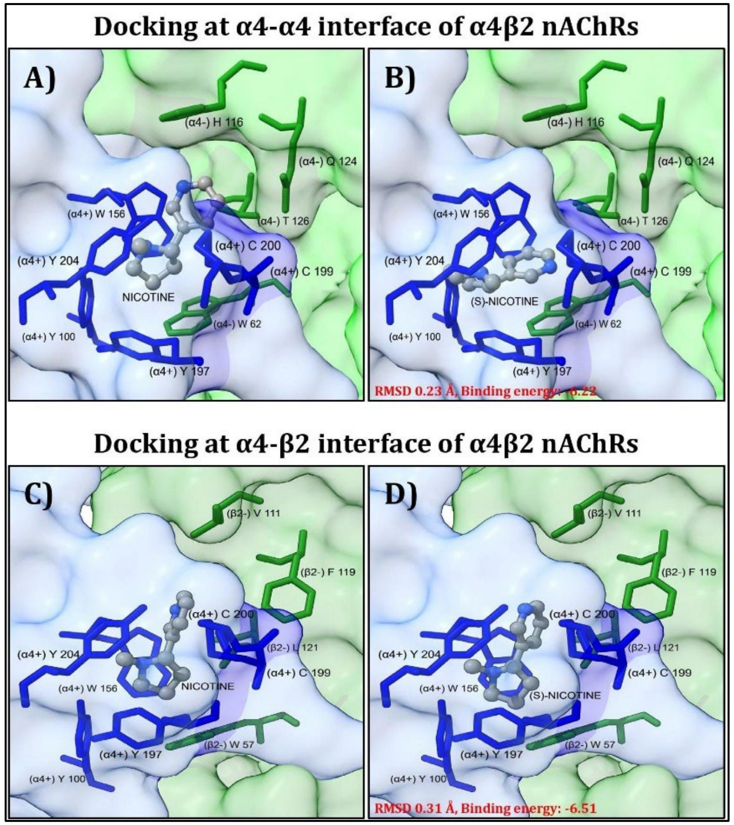

Supplementary Figure S4. The native position of NIC molecule (**A, C**) and the best theoretical binding pose of (S)-NIC (**B, D**) in the α4-α4 interface (top panel) and α4-β2 interface (bottom panel) of α4β2 nAChRs (3α:2β stoichiometry).

Supplementary Table S3. The binding energies, ligand efficiencies and the number of hydrogen (H) bonds formed for the best three binding positions of the ligands in the corresponding receptor

Receptor		Ligand	Binding energy	Ligand efficiency	H bonds formed
α9 nAChRs	α9-α9 interface	(S)-Nicotine	-7.12	-0.59	0
			-6.07	-0.56	0
			-5.51	-0.46	0
		(S)-6-Hydroxynicotine	-7.38	-0.57	1
			-7.37	-0.57	1
			-7.28	-0.56	1
α3β2α5 nAChRs (2:2:1 stoichiometry)	α3-β2 interface	(S)-Nicotine	-6.48	-0.54	0
			-6.48	-0.54	0
			-6.45	-0.54	0
		(S)-6-Hydroxynicotine	-6.69	-0.51	1
			-6.67	-0.51	1
			-6.52	-0.5	1
	α5-α3 interface	(S)-Nicotine	-5.71	-0.48	0
			-5.71	-0.48	0
			-5.71	-0.48	0
		(S)-6-Hydroxynicotine	-6.28	-0.48	1
			-6.27	-0.48	1
			-6.24	-0.48	1
α3β4α5 nAChRs (2:2:1 stoichiometry)	α3-β4 interface	(S)-Nicotine	-6.07	-0.51	0
			-6.06	-0.51	0
			-6.06	-0.51	0
		(S)-6-Hydroxynicotine	-6.55	-0.5	0
			-6.53	-0.5	0
			-6.52	-0.5	0
	α5-α3 interface	(S)-Nicotine	-5.82	-0.49	1
			-5.78	-0.48	1
			-5.75	-0.48	1
		(S)-6-Hydroxynicotine	-6.05	-0.47	2
			-6.02	-0.46	2
			-6	-0.46	1

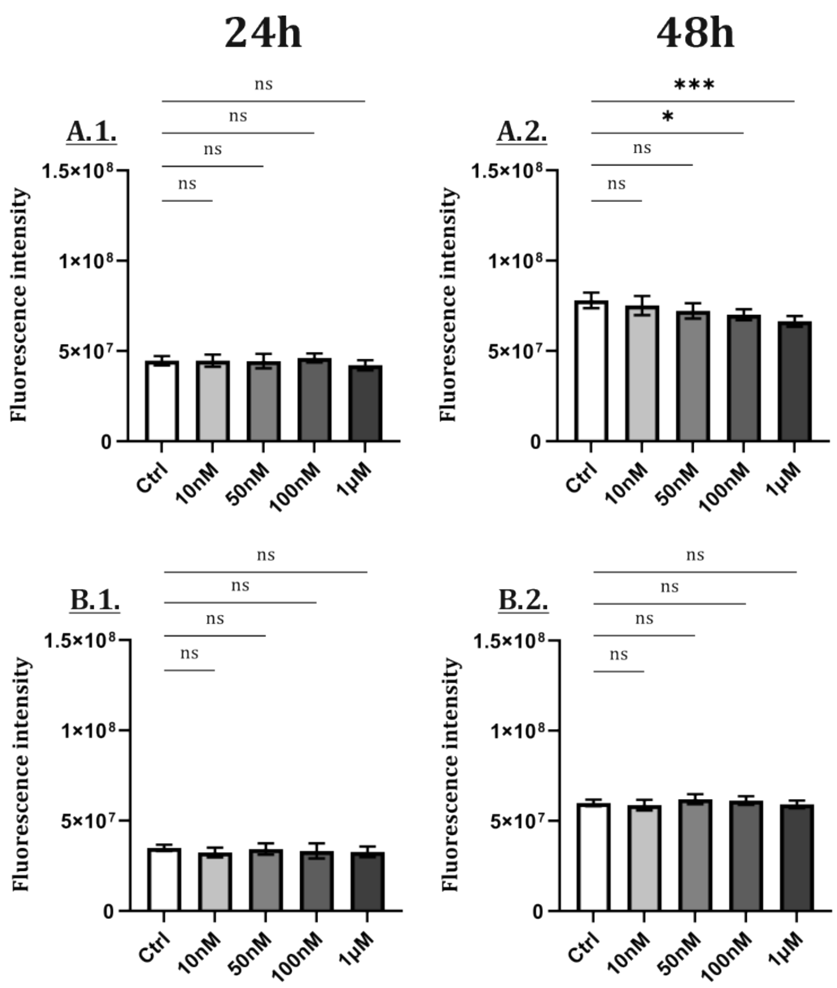

Supplementary Figure S5. Cellular viability 24 and 48 hours post incubation of 6HLN at different concentrations (10nM, 50nM, 100nM, and 1μM) in (**A**) 16HBE14o and (B) MCF10A normal cell lines. Values are means ± S.D. (n = 5). ns - non-significant, * $p \leq 0.05$; *** $p \leq 0.001$.

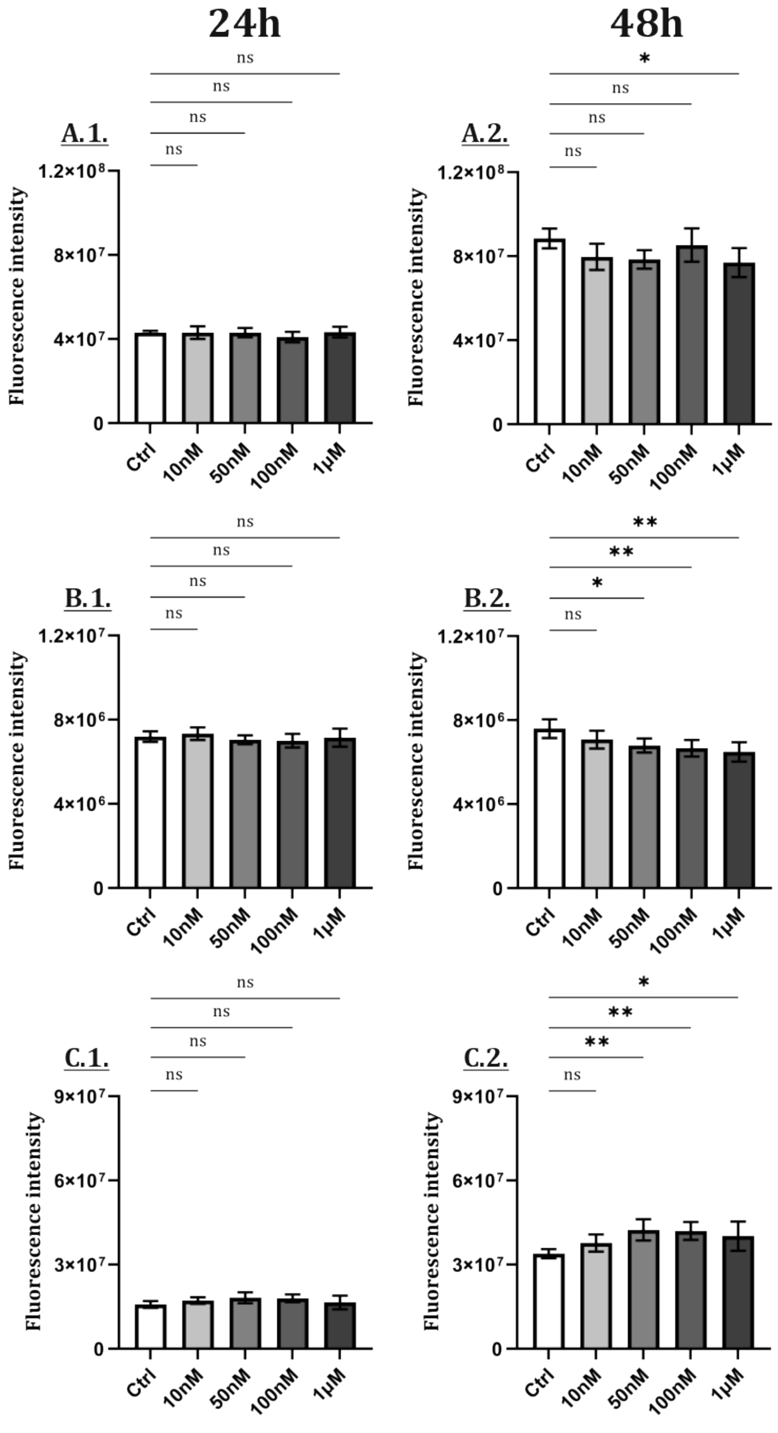

Supplementary Figure S6. Cellular viability 24 and 48 hours post incubation of 6HLN at different concentrations (10nM, 50nM, 100nM, and 1µM) in (A) A549, (B) MCF-7, and (C) U87 cancer cell lines. Values are means ± S.D. (n = 5). ns - non-significant, * $p \leq 0.05$; ** $p \leq 0.01$.

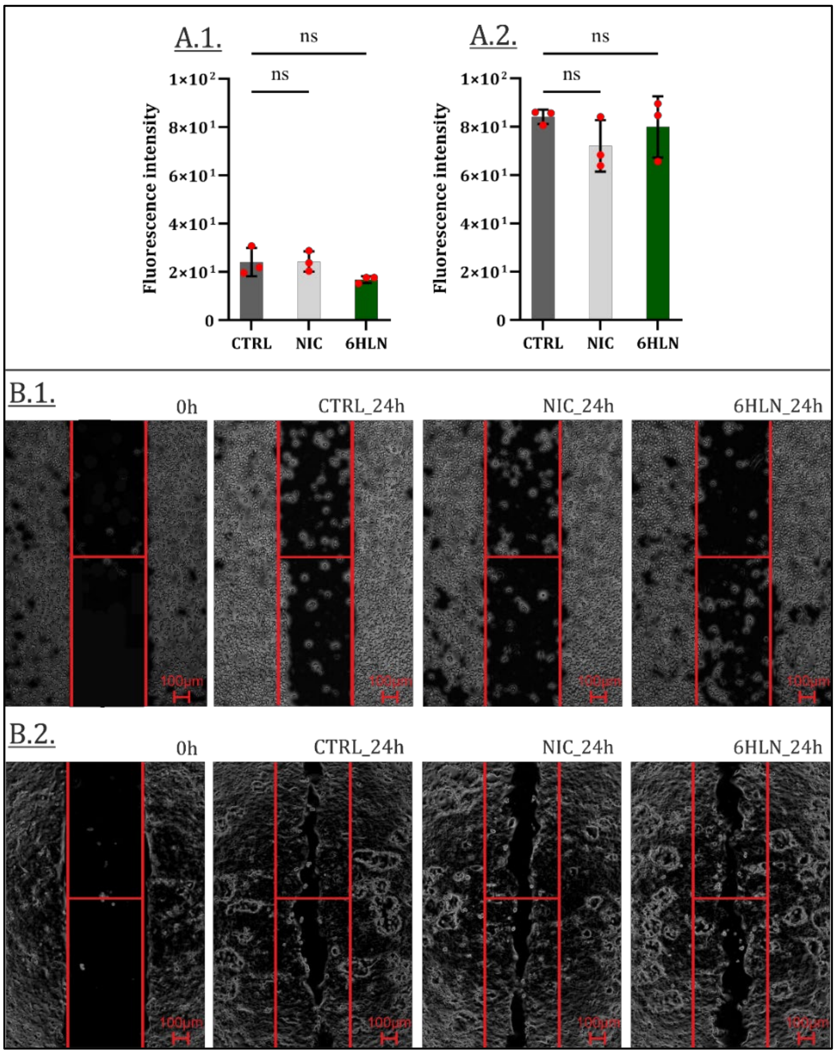

Supplementary Figure S7. Wound closure 24 hours post incubation of NIC / 6HLN (50nM) in 16HBE14o (A.1.) and MCF10A (A.2.) cells. B – Representative pictures: 16HBE14o (B.1.) and MCF10A (B.2.) cells. Values are means ± S.D. (n = 3).
